# A human proteomic dataset from untreated and depleted/enriched serum samples

**DOI:** 10.1016/j.dib.2018.06.042

**Published:** 2018-06-26

**Authors:** Salvatore Pisanu, Grazia Biosa, Laura Carcangiu, Sergio Uzzau, Daniela Pagnozzi

**Affiliations:** Porto Conte Ricerche, Science and Technology Park of Sardinia, Alghero, Sassari, Tramariglio, Italy

## Abstract

We present a proteomic dataset generated from a human serum sample and the enriched/depleted fractions obtained by seven commercial products. This report is related to the research article entitled “Comparative evaluation of seven commercial products for human serum enrichment/depletion by shotgun proteomics” [1]. All samples were analyzed by LC-MS/MS, label free quantitation using the spectral counting approach, and Gene Ontology (GO) annotation. Protein relative abundances and functions were reported.

**Specifications Table**

**Table:** 

Subject area	Biology
More specific subject area	Proteomics
Type of data	A. Tables with all identified proteins, and peptides.
	B. Protein relative abundance (NSAF), and Gene Ontology annotation.
How data was acquired	Q Exactive mass spectrometer interfaced with an UltiMate 3000 RSLCnanoLC system (Thermo Fisher Scientific)
Data format	xlsx file (Excel tables)
Experimental factors	Serum highly abundant protein depletion by seven commercial products
Experimental features	A. Serum depletion/enrichment
	B. Filter-aided sample preparation (FASP)
	C. Mass spectrometry analysis (LC-MS/MS)
	D. Label free quantitation
	E. Gene Ontology annotation
Data source location	Tramariglio, Alghero (Sassari), Italy
Data accessibility	Data is within this article, provided as supplementary file
Related research article	S. Pisanu, G. Biosa, L. Carcangiu, S. Uzzau, D. Pagnozzi. Comparative evaluation of seven commercial products for human serum enrichment/depletion by shotgun proteomics, Talanta 185, 2018, 213-220. 10.1016/j.talanta.2018.03.086

**Value of the data**

• Proteomic analysis of untreated and depleted/enriched human serum samples was generated by a wide selection of commercially available kits.
• Dataset includes 1266 and 9905 non-redundant proteins and peptides, respectively.
• Relative abundance and GO annotation data might be a useful support for other researchers to select the method of choice according to the target of interest.

## 1 Data

Peptide and protein identifications from a human serum sample and from the depleted/enriched fractions obtained after treatment with seven commercial kits are reported, with the corresponding peptide spectrum matches (PSMs). Moreover, protein abundances and gene ontology annotation, including biological process, molecular function and cellular component are showed. Data are provided as Supplementary file.

## 2 Experimental design, materials, and methods

### 2.1 Sample treatment

Human serum sample was either treated by seven commercial products (Top 2 Abundant Protein Depletion Spin Columns “Top 2”, Top 12 Abundant Protein Depletion Spin Columns “Top 12”, Albumin and IgG Depletion SpinTrap “SpinTrap”, Qproteome Albumin/IgG Depletion “Qproteome”, ProteoPrep Immunoaffinity Albumin and IgG Depletion Kit “ProteoPrep”, Albumin/IgG Removal “CibB-A”, and ProteoMiner beads “ProteoMiner”), or analyzed as untreated sample. Two technical replicates were performed for each procedure. Depletions were performed according to the protocols and the sample volumes recommended by the manufacturers. Then, all samples were processed with the filter-aided sample preparation (FASP) protocol, with minor modifications, as described by Pisanu et al. [1].

### 2.2 LC-MS/MS analysis

Mass spectrometry analysis was carried out on a Q Exactive interfaced with an UltiMate 3000 RSLCnanoLC system (Thermo Fisher Scientific, San Jose, CA, USA), as previously described by Pagnozzi et al. [2] with some adjustments [1]. Runs were performed loading 4 μg of peptide mixture of each sample, using a linear gradient of 245 min. The mass spectrometer was set up in a data dependent MS/MS mode, with Higher Energy Collision Dissociation as the fragmentation method. Peptide identification was performed using Proteome Discoverer (version 1.4; Thermo Scientific) as described by Pisanu et al. [1].

### 2.3 Label-free quantitation and Gene Ontology

Protein relative abundance was evaluated by spectral counting (SpC) approach [3], and for each protein the normalized spectral abundance factor (NSAF) was calculated according to Old et al. [4]. Protein identification data were subjected to Gene Ontology (GO) annotation for biological process, molecular function, and cellular component using UniProt Knowledgebase (UniProtKB) database in Perseus software (v.1.6.0.7) [5], [6].
